# Correction: Implementation barriers and facilitators to a COVID-19 intervention in Bangladesh: The benefits of engaging the community for the delivery of the programme

**DOI:** 10.1186/s12913-023-09030-5

**Published:** 2023-01-20

**Authors:** Fahmida Akter, Malika Tamim, Avijit Saha, Imran Ahmed Chowdhury, Omor Faruque, Animesh Talukder, Mohiuddin Ahsanul Kabir Chowdhury, Monzur Morshed Patwary, Albaab-Ur Rahman, Morseda Chowdhury, Malabika Sarker

**Affiliations:** 1 BRAC James P Grant School of Public Health, BRAC University, Dhaka, Bangladesh; 2 Health, Nutrition, and Population Program, BRAC, Dhaka, Bangladesh; 3 Heidelberg Institute of Global Health, Heidelberg University, Heidelberg, Germany

**Correction: BMC Health Serv Res 22, 1590 (2022)**

**https://doi.org/10.1186/s12913-022-08939-7**

Following publication of the original article [1], the figure captions of Fig. 1, Fig. 2 and Fig. 3 need to be updated, and several figure citations should be corrected.

In addition, the sentence “The project duration was too short for all the activities undertaken.” (Senior management, BRAC, KII 50) under the section ‘The short duration of the project’ should be formatted as a quotation, not a heading.

The original article [1] has been updated.
